# Change in commute mode and body-mass index: prospective, longitudinal evidence from UK Biobank

**DOI:** 10.1016/S2468-2667(16)30006-8

**Published:** 2016-12

**Authors:** Ellen Flint, Elizabeth Webb, Steven Cummins

**Affiliations:** a Department of Social and Environmental Health Research, London School of Hygiene & Tropical Medicine, London, UK; b International Centre for Lifecourse Studies, Department of Epidemiology and Public Health, University College London, London, UK

## Abstract

**Background:**

Insufficient physical activity is a determinant of obesity and cardiovascular disease. Active travel to work has declined in high-income countries in recent decades. We aimed to determine which socioeconomic and demographic characteristics predicted switching to or from active commuting, whether switching from passive to active commuting (or the reverse) independently predicts change in objectively measured body-mass index (BMI), and to ascertain whether any association is attenuated by socioeconomic, demographic, or behavioural factors.

**Methods:**

This study used longitudinal data from UK Biobank. Baseline data collection occurred at 22 centres between March, 2006, and July, 2010, with a repeat assessment at one centre (Stockport) between August, 2012, and June, 2013, for a subset of these participants. Height and weight were objectively measured at both timepoints. We included individuals present at both timepoints with complete data in the analytic sample. Participants were aged 40–69 years and commuted from home to a workplace on a regular basis at both baseline and follow-up. Two exposures were investigated: transition from car commuting to active or public transport commuting and transition from active or public transport to car commuting. Change in BMI between baseline and repeat assessment was the outcome of interest, assessed with bivariate and multivariate logistic regression models.

**Findings:**

502 656 individuals provided baseline data, with 20 346 participating in the repeat assessment after a median of 4·4 years (IQR 3·7–4·9). 5861 individuals were present at both timepoints and had complete data for all analytic variables. Individuals who transitioned from car commuting at baseline to active or public transportation modes at follow-up had a decrease in BMI of −0·30 kg/m^2^ (95% CI −0·47 to −0·13; p=0·0005). Conversely, individuals who transitioned from active commuting at baseline to car commuting at follow-up had a BMI increase of 0·32 kg/m^2^ (0·13 to 0·50; p=0·008). These effects were not attenuated by adjustment for hypothesised confounders. Change in household income emerged as a determinant of commute mode transitions.

**Interpretation:**

Incorporation of increased levels of physical activity as part of the commute to work could reduce obesity among middle-aged adults in the UK.

**Funding:**

UK Medical Research Council.

## Introduction

Mid-life is a key stage for the development of obesity and cardiovascular disease risk.^1^ In England, 78% of men and 65% of women aged 45–75 years are overweight or obese,^1^ and 44% of adults aged 55–64 years do not meet recommended levels of physical activity.^2^ In the past 50 years, mass adoption of private motorised transport and the modification of built environments to facilitate car use has coincided with a decline in active travel and a rise in population prevalence of overweight and obesity. Laverty and colleagues^3^ reported that adults aged 50–65 years were 55% less likely to commute via public transport, 45% less likely to commute on foot, and 30% less likely to commute by bicycle than were 16–29-year olds. The commute to work has been identified by the UK National Institute for Health and Care Excellence (NICE) as a key intervention point.^4^ In England and Wales, 23·7 million working-age individuals commute regularly to a workplace, with 67% travelling by car.^5^ For many, a transition to more active modes might be possible, without requiring unacceptable time or financial costs.

Previous studies^3^, ^6^, ^7^, ^8^, ^9^ have found a strong, independent, cross-sectional association between active or public transport commuting and reduced obesity risk. Compared with car commuters, individuals who used active and public transport had lower body-mass indexes (BMIs) and percentage body fat, and lower rates of diagnosed diabetes and hypertension.^3^, ^6^, ^7^, ^9^ A graded effect has also been found, whereby the magnitude of effect is greater across successively more active transport modes.^9^ However, a limitation of the evidence has been an overreliance on cross-sectional data, limiting causal inference. Martin and colleagues^10^ used longitudinal data from the British Household Panel Study to show that commuters who switched from car commuting to active or public modes experienced a significant, independent reduction in self-reported BMI. Equally those who transitioned from active to car commuting reported a significant increase in BMI. Mytton and colleagues^11^ used two waves of data from the Commuting in Cambridge study to demonstrate that maintenance of cycle commuting was associated with lower BMI when compared with maintenance of sedentary commuting. However corroborative results were not found for walking.^11^ The protective effects of cycle commuting have also been reported for schoolchildren.^12^ A Norwegian study^13^ showed that maintenance of active travel in pregnancy predicted lower gestational weight gain than switching to more sedentary modes. Much of the present evidence-base is hampered by reliance on self-reported height and weight, which are prone to bias.^14^ Besides changing of residential or employment locations, evidence is lacking on what socioeconomic, demographic, and health-related factors predict transitions from car to active commuting, or vice versa.

**Research in context**

**Evidence before this study**

Studies have repeatedly shown that active commuting to work contributes to greater overall physical activity and is associated with reduced body-mass index (BMI), percentage body fat, and risk of reporting hypertension and type 2 diabetes diagnoses. Previous work has shown a graded effect of active commuting on BMI, wherein greater magnitudes of association are observed across progressively more active transportation modes. However, much of the existing evidence base is hampered by cross-sectional study designs and self-reported health outcome data. These limitations make causality hard to establish, and accuracy difficult to ensure. Previous studies using longitudinal data have contributed valuable evidence by showing that BMI decreases as individuals transition to, or maintain, active commuting.

**Added value of this study**

This longitudinal study builds on these foundations by using objectively measured height and weight to derive an objective change in BMI outcome. The dataset, UK Biobank, allows for a focus on a lifecourse stage during which individuals are at particularly high risk for development of obesity and its behavioural risk factors: mid-life. The study shows that switching from more active (walking, cycling, or public transport) to more passive (car) commuting independently predicted a significant decrease in BMI of about 0·3 kg/m^2^. Conversely, switching from passive to more active commuting significantly and independently predicted a BMI increase of the same magnitude. Change in household income was found to be the key driver of commute mode transitions.

**Implications of all the available evidence**

Active commuting is a significant, independent determinant of bodyweight in mid-life. Public health policies that promote active travel to work, through encouragement of walking, cycling, and the use of public transport, could help prevent obesity in this critical period of the lifecourse (age 40–69 years).

In this study, we used longitudinal data from UK Biobank, a large population-based study of UK adults in mid-life, to investigate associations between changing commute mode and objective measures of BMI, and to identify the determinants of transitions to more active modes of commuting. We aimed to determine which socioeconomic and demographic characteristics predicted switching to or from active commuting, investigate whether switching from car to active commuting (or the reverse) independently predicts change in objectively measured BMI, and to ascertain whether any association is attenuated by socioeconomic, demographic, or behavioural factors.

## Methods

### Study design and data collection

We used survey data from UK Biobank (project 5935) to longitudinally study adults aged 40–69 years, selected via National Health Service (NHS) patient registers and recruited to 22 regional assessment centres. Biobank collected baseline data nationwide between March, 2006, and July, 2010. The project also did repeat assessment at a single location (Stockport UK Biobank Coordinating Centre) between August, 2012, and June, 2013, for a subset of these participants.

The sample of individuals who were present at both baseline and follow-up was refined to include only participants with complete data for all analytic variables at both timepoints. Four analytic samples were derived to address three objectives. For objective 1, we assessed individuals who had complete data for all hypothesised predictors and had either experienced a transition from car to active or public transport or conversely a transition from active or public transport to car commuting. For objective 2, we assessed individuals who experienced a transition from car to active or public transport or remained car commuters, and had complete data for all covariates. For objective 3, we assessed individuals who experienced a transition from active or public transport to car commuting or remained public or active transport users, and had complete data for all covariates.

UK Biobank has approval from the North West Multi-centre Research Ethics Committee, the Patient Information Advisory Group, and the Community Health Index Advisory Group. Further details on the rationale, study design, survey methods, data collection, and ethical approval are available elsewhere.^15^, ^16^, ^17^, ^18^

### Procedures

At both timepoints, participants were asked “what types of transport do you use to get to and from work?” and were able to select one or more of the following mode categories: car or motor vehicle, walk, public transport, or cycle. Responses were dichotomised to create a binary variable indicating whether the individual commuted solely by car, or by any other mode or mix of modes. This result was then used to derive two binary variables indicating whether the respondent had experienced one of the following transitions between baseline and repeat assessment: transition from car commuting to active or public transport commuting or transition from active or public transport to car commuting. These variables were used as outcome variables in the analyses for objective 1, and exposure variables for objectives 2 and 3.

### Outcomes

Change in BMI between baseline and follow-up was the primary outcome for study objectives 2 and 3. Anthropometric measurements were taken by trained staff using standard procedures detailed elsewhere.^18^ Height (measured using the Seca 202 stadiometer (Seca; Birmingham, UK) and weight (Tanita BC-418MA body composition analyser (Tanita; Amsterdam, Netherlands), was used to derive BMI via the standard formula. Change in BMI was calculated for each individual by subtracting BMI at baseline from BMI at follow-up.

### Covariates

Factors hypothesised to confound the association between commute mode transition and BMI change were adjusted for in statistical analyses. They comprised both time-invariant factors (fixed characteristics or baseline measurements) and time-varying factors (changes between baseline and follow-up). Hypothesised time-invariant confounders were age at baseline, sex, ethnicity (white British, other white background, south Asian, Black Caribbean, Black African, Chinese, mixed ethnicity, other), baseline BMI, baseline highest educational qualification (university or college degree, further education [A level or equivalent], higher secondary education [ordinary level, GCSEs, or equivalent], secondary education [CSEs or equivalent], vocational qualifications [NVQ, Higher National Diploma, Higher National Certificate, or equivalent], professional qualifications, or none), baseline gross annual household income category (<£18 000, £18000–30 999, £31 000–51 000, £52 000–100 000, or >£100 000), manual occupation at baseline (usually or always *vs* rarely or never), baseline job involves standing or walking (usually or always *vs* rarely or never); and baseline days per week of at least 10 min moderate leisure physical activity. Hypothesised time-varying confounders between baseline and follow-up were change in income category (stable, decrease, or increase), self-rated general health (stable good health, good to poor health transition, poor to good health transition, or stable poor health), manual occupation status (stable, transition to non-manual work, or transition to manual work), days per week of moderate physical activity (stable, decrease, or increase), and occupational standing or walking level (stable, decrease, or increase).

### Statistical analysis

The analytic sample size was large enough to produce reliable estimates of BMI change. We used descriptive analysis to identify the prevalence of switching from active to sedentary modes of commuting and to describe the distribution of other key variables. We fitted separate bivariate logistic regression models to identify which socioeconomic, demographic, health, and behavioural factors predicted a transition to or from car commuting. To assess effects on BMI, we used two series of nested multivariate linear regression models to investigate the effects of switching from car commuting to active or public modes and to investigate the effects of switching from active or public commuting modes to car commuting. In each series of nested models, model 1 tested for a bivariate association between the commute transition exposure and the obesity outcome. Demographic and socioeconomic covariates were added for model 2 (baseline BMI, age, sex, ethnicity, baseline household income, household income change, and educational attainment). For the final model (model 3), health, physical activity, and occupational covariates were added (self-rated general health transitions, manual occupation transitions, days per week of leisure moderate physical activity, and changes between baseline and follow-up, occupational physical activity transitions). All analyses were done with Stata/SE, version 14.

### Role of the funding source

The sponsors had no role in the design of the study; collection, analysis, and interpretation of the data; or writing of the report. The corresponding author had full access to the data and responsibility for the decision to submit for publication.

## Results

502 656 adults were surveyed at baseline. 20 346 (21%) participated in the repeat assessment (median follow-up 4·4 years [IQR 3·7–4·9]).^18^ 5861 individuals had complete data for all analytic variables at both baseline and follow-up (table 1). For objective 1, 2993 individuals with complete data transitioned from car to active or public transport (table 2) and 1277 individuals with complete data transitioned from active or public transport to car commuting (table 2). For objective 2, 4126 individuals with compete covariate data transitioned from car to active or public transport or remained car commuters (table 3). For objective 3, 1735 individuals with compete covariate data transitioned from active or public transport to car commuting or remained public or active transport users (table 4).

3646 baseline car commuters remained car commuters at follow-up (table 1). However, 480 (8%) individuals switched to active or public modes of commuting. Of these individuals, 44 (9%) had switched from car to exclusive walking or cycling, with 436 (91%) using public transport for part of their journey. Conversely, 416 (7%) individuals who commuted by active public modes at baseline had switched to car commuting at follow-up. Of these, 33 (8%) switched from exclusive walking or cycling with the rest switching from public transport. 1319 individuals used active or public modes at both baseline and follow-up. 1011 (17%) of 5861 had a decline in their household income category between baseline and follow-up, while a similar proportion reported an increase (18%, table 1). Although 4421 (75%) reported good general health at both timepoints, 616 (11%) had poor health at both timepoints and 490 (8%) went from good to poor general health.

Only income was consistently associated with commuting mode transitions, for both sexes (table 2). Compared with individuals who remained in the same income category at both timepoints, respondents who experienced income loss were more likely to transition from car commuting to active or public modes (unadjusted OR 1·34, 95% CI 1·02–1·76; p=0·033). However, respondents who experienced income loss were also more likely to report a transition from active to public transport commuting to car travel compared with those who were active or public mode users at both timepoints (1·46, 1·04–2·05; p=0·0280). These results probably reflect changes in occupation, which might explain both income category and commute mode. Indeed, experiencing an income category increase was also predictive of transitioning from active or public modes to car use (1·62, 1·17–2·24; p=0·0040).

The 480 individuals who switched from car commuting at baseline to active or public commuting modes at follow-up were compared with the 3646 individuals who remained car commuters at both timepoints (table 3). In the fully adjusted model, a transition to a more active commute was significantly and independently predictive of a 0·30 kg/m^2^ decrease in BMI (95% CI −0·47 to −0·13; p=0·0005).

The 416 individuals who switched from active or public commuting modes at baseline to car commuting at follow-up were compared with the 1319 individuals who reported commuting via active or public modes at both timepoints (table 4). In the fully adjusted model, experiencing a transition to car commuting was significantly and independently predictive of a 0·32 kg/m^2^ increase in BMI (95% CI 0·13 to 0·50; p=0·0008). Adjustment for hypothesised time varying and time invariant confounders did not attenuate the effects of commute mode transition.

## Discussion

In our comparison of BMI changes in middle-aged adults who switched mode of commute with their counterparts who maintained their mode of commute, individuals who transitioned from car commuting at baseline to using active or public modes at follow-up had an average BMI decrease of about 0·3 kg/m^2^. This effect was not attenuated by adjustment for hypothesised demographic, socioeconomic, health, and behavioural confounders. The inverse effect was also found: individuals who transitioned from active or public modes at baseline to car commuting at follow-up typically had a BMI increase of about 0·3 kg/m^2^. This effect was also independent of fixed or changing demographic, socioeconomic, health, and behavioural factors. Of these factors, only income emerged as a consistent, independent predictor of commute mode transition. For the average man in the baseline sample (aged 52 years, 176·6 cm tall, weighing 85·1 kg) a BMI decrease of 0·3 kg/m^2^ is equivalent to a weight loss of approximately 1·0 kg (2·2 lbs). For the average woman in the baseline sample (aged 51 years, 163·9 cm tall, weighing 70·0 kg) a BMI decrease of 0·3 kg/m^2^ is equivalent to a weight loss of approximately 0·8 kg (1·8 lbs). Economic modelling undertaken to inform NICE obesity guidelines^19^ suggested that for overweight adults, weight management interventions costing £100 or less per head are cost-effective for the NHS if they result in a maintained weight loss of at least 1 kg.

Most transitions reported here were between car use and public transport use, and vice versa. Previous cross-sectional studies have shown that compared with car commuting, public transport independently and significantly predicts lower BMI, with similar effect sizes to active modes.^3^, ^6^ However in previous work using cross-sectional data from UK Biobank’s baseline sample,^9^ although public transport commuting predicted lower BMI than car use, cycling and to a lesser extent walking to work was associated with lower BMI scores than public transport. Therefore, findings from the present study are probably an underestimation of the BMI decrease one would expect if it had been possible to model transitions between, for example, car commuting and cycle commuting. However this study adds strength to the argument that the incidental physical activity associated with the use of public transport, such as walking to and from transit stops, might play an important part in obesity prevention.

These results support and corroborate the findings of Martin and colleagues^10^ in showing an independent, significant association between switching between sedentary and active commute modes and BMI change in the British Household Panel Survey 2004–07. Effect sizes are strikingly consistent: they found that switching from car to active or public transport commuting predicted a decrease in self-reported BMI of 0·32 kg/m^2^ (95% CI −0·60 to −0·05). They also found that the opposite transition predicted a BMI increase of 0·34 kg/m^2^ (0·05 to 0·64). Together these two studies provide strong evidence for an association between active commuting and BMI.

Our study has strengths and limitations. UK Biobank is a high quality data resource that allows the use of objectively measured height and weight to provide unbiased BMI data. The comprehensive dataset also allows adjustment for a wide range of time-varying and time-invariant confounders. As randomised controlled trials are difficult to do in this area of research, longitudinal observational data might represent the best available evidence for policy development. However, the study is also subject to a range of limitations, many stemming from the relatively constrained sample size. First and foremost is the loss of nuance created by the need to combine active modes with public transport modes. This combination was attributable to the low prevalence of walking and cycling and the even lower incidence of transitions involving walkers and cyclists. Most respondents who switched from car commuting transitioned to public transport rather than to walking or cycling (and vice versa). The effect sizes reported in this study are therefore expected to be an underestimation of the likely BMI effects of transitions to walking or cycling. The necessary exclusion of commute distance from analyses is a limitation of this study, and a source of effect underestimation for long distance walkers or cyclists. The precise point at which a mode transition occurred is not known, and duration of exposure to a new commute mode is likely to be heterogeneous. As a result of these limitations, the precise effect sizes for the association between commute mode transitions and BMI change are subject to uncertainty.

Residual confounding by factors such as menopausal status, dietary energy intake, and physical activity might have occurred. Although we adjusted for leisure, occupational, and non-commute travel physical activity, these variables were self-reported and not comprehensive.

Thus, although this study benefits from the inclusion of an objectively measured health outcome, the use of a self-reported exposure is a limitation. Social desirability bias might lead to under-reporting of car commuting. However, individuals’ propensity to misreport mode is likely to remain relatively fixed over time, strengthening the internal validity of the study.

The study is also subject to limitations stemming from attrition (mostly due to retirement) and missing data. Individuals who dropped out of the study could be systematically different from those who contributed to both waves of data collection. By definition, sample members with data at both timepoints were in the Stockport assessment centre catchment area. This may limit the generalisability of results to this geographical area. Only 21% of those invited by UK Biobank to take part in the repeat assessment did so, as described in the UK Biobank Repeat Assessment documentation.^18^ Furthermore, UK Biobank is not strictly representative of the UK mid-life population so results might not be fully generalisable.

This study shows that individuals who switched from car commuting to public transport or active modes experienced a decrease in BMI. This decrease was independent of changes in the socioeconomic, demographic, health, and behavioural factors observed over the same period. These findings suggest that policies that enable and encourage the maintenance and uptake of commuting by more active modes such as public transportation, walking, or cycling could have an effect on obesity prevalence in this high-risk age group. Only 896 (15%) individuals in this study had a commute mode transition, suggesting untapped potential exists for interventions to facilitate uptake of active or public transport. Most individuals who switched from car commuting transitioned to public transport. The effects observed in this study are therefore primarily related to mass transit and the benefits gained from the incidental physical activity associated with its use. Thus, this study is likely to underestimate the effects on BMI of walking or cycling to work. Efforts to increase active travel to work through widening of access to mass transit systems and integrating them with opportunities for walking and cycling might represent an effective policy response to the obesity epidemic.

## Figures and Tables

**Table 1:** Descriptive analysis

		**Respondents (n=5861)**
Baseline age (years)		51 (6·25)
Baseline BMI (kg/m^2^)		26·67 (4·47)
Days per week of ≥10 min moderate physical activity		3·22 (2·27)
Car commuter at t0 and t1		3646 (62%)
Commuted by active or public commute modes at t0 and t1		1319 (23%)
Transitioned from car to active or public modes, t0 to t1		480 (8%)
Transitioned from active or public modes to car, t0 to t1		416 (7%)
Sex		
	Male	2977 (51%)
	Female	2884 (49%)
Ethnicity		
	White British	5293 (90%)
	Other white background	388 (7%)
	South Asian	47 (1%)
	Black Caribbean	18 (<1%)
	Black African	22 (<1%)
	Chinese	21 (<1%)
	Mixed background	28 (<1%)
	Other ethnic background	44 (1%)
Gross annual household income		
	<£18 000	406 (7%)
	£18 000–£30 999	1158 (20%)
	£31 000–£51 999	1892 (32%)
	£52 000–£100 000	1932 (33%)
	>£100 000	473 (8%)
Gross annual household income category change, t0 to t1		
	Stable	3793 (65%)
	Decrease	1011 (17%)
	Increase	1057 (18%)
Highest educational qualification at baseline		
	College or university degree	2919 (50%)
	A levels or equivalent	803 (14%)
	O levels/GCSEs or equivalent	1083 (18%)
	CSEs or equivalent	287 (5%)
	NVQ, HND, HNC, or equivalent	338 (6%)
	Other professional qualifications	229 (4%)
	None of the above qualifications	202 (3%)
Self-rated health transition between t0 and t1		
	Stable good health	4421 (75%)
	Good to poor health	490 (8%)
	Poor to good health	334 (6%)
	Stable poor health	616 (11%)
Manual work status		
	Non-manual work	4209 (72%)
	Manual work	1652 (28%)
Change in manual work status between t0 and t1		
	Stable	5193 (89%)
	Transition to non-manual work	359 (6%)
	Transition to manual work	309 (5%)
Change in days per week of ≥10min moderate activity, t0 to t1		
	Stable	1670 (28%)
	Decrease	2012 (34%)
	Increase	2179 (37%)
Job involves standing or walking		
	Never/rarely	2456 (42%)
	Sometimes	1878 (32%)
	Usually/always	1527 (26%)
Change in occupational standing or walking levels, t0 to t1		
	Stable	4226 (72%)
	Decrease	832 (14%)
	Increase	803 (14%)

Data are mean (SD) or n (%). t0=baseline. t1=repeat assessment.

**Table 2:** Separate bivariate logistic regression models assessing associations between demographic, socioeconomic, health, and behavioural factors for individuals transitioning from car commuting to active or public mode commuting between baseline (t0) and follow-up (t1; n=2993) or vice versa (n=1277)

		**From car to active or public mode commuting (n=2993)**		**From active or public mode to car commuting (n=1277)**	
		Unadjusted OR (95% CI)	p value	Unadjusted OR (95% CI)	p value
Baseline BMI		0·98 (0·96–1·01)	0·17	1·04 (1·01 to 1·07)	0·0050
Baseline age (years)		0·99 (0·98–1·01)	0·57	0·99 (0·97 to 1·01)	0·34
Sex					
	Male	1 (reference)		1 (reference)	
	Female	1·09 (0·88–1·36)	0·42	0·97 (0·75 to 1·25)	0·82
Highest educational qualification at baseline					
	College or university degree	1 (reference)		1 (reference)	
	A levels or equivalent	0·88 (0·64–1·21)	0·42	1·64 (1·14 to 2·48)	0·0080
	O levels/GCSEs or equivalent	0·64 (0·47–0·88)	0·0060	1·74 (1·23 to 2·47)	0·0020
	CSEs or equivalent	1·10 (0·70–1·75)	0·68	1·96 (0·96 to 4·03)	0·070
	NVQ, HND, HNC, or equivalent	0·38 (0·21–0·72)	0·0030	1·25 (0·68 to 2·31)	0·47
	Other professional qualifications	0·50 (0·27–0·94)	0·033	0·82 (0·31 to 2·19)	0·69
	None of the above qualifications	0·56 (0·27–1·16)	0·12	0·72 (0·24 to 2·10)	0·55
Gross annual household income					
	<£18 000	1 (reference)		1 (reference)	
	£18 000–£30 999	0·90 (0·54–1·51)	0·69	1·48 (0·85 to 2·59)	0·17
	£31 000–£51 999	0·87 (0·53–1·42)	0·58	1·46 (0·86 to 2·50)	0·16
	£52 000–£100 000	0·70 (0·43–1·15)	0·16	1·64 (0·96 to 2·78)	0·070
	>£100 000	0·98 (0·56–1·74)	0·96	1·45 (0·74 to 2·84)	0·28
Gross annual household income category change, t0 to t1					
	Stable	1 (reference)		1 (reference)	
	Decrease	1·34 (1·02–1·76)	0·033	1·46 (1·04 to 2·05)	0·0280
	Increase	1·08 (0·81–1·44)	0·60	1·62 (1·17 to 2·24)	0·0040
Self-rated health transition between t0 and t1					
	Stable good health	1 (reference)		1 (reference)	
	Good to poor health	1·15 (0·79–1·67)	0·48	1·26 (0·80 to 1·98)	0·32
	Poor to good health	1·24 (0·78–1·96)	0·36	0·84 (0·48 to 1·48)	0·55
	Stable poor health	0·77 (0·51–1·16)	0·21	1·50 (0·95 to 2·37)	0·080
Days per week of ≥10 min moderate physical activity		0·99 (0·95–1·04)	0·82	0·93 (0·88 to 0·99)	0·0110
Walking for pleasure		0·98 (0·91–1·06)	0·64	0·99 (0·90 to 1·09)	0·87
Baseline manual work status					
	Non-manual work	1 (reference)		1 (reference)	
	Manual work	0·80 (0·62–1·02)	0·070	1·20 (0·89 to 1·61)	0·24
Change in occupational standing or walking levels, t0 to t1					
	Stable	1 (reference)		1 (reference)	
	Decrease	0·89 (0·65–1·22)	0·48	1·11 (0·76 to 1·62)	0·59
	Increase	1·00 (0·73–1·37)	0·99	1·33 (0·93 to 1·91)	0·12

**Table 3:** Nested multivariate linear regression models testing whether experiencing a transition from car commuting to active or public mode commuting between baseline and follow independently predicted body-mass index change (n=4126)

		**Model 1**		**Model 2**		**Model 3**	
		Coefficient (95% CI)	p value	Coefficient (95% CI)	p value	Coefficient (95% CI)	p value
Stable car user		0		0		0	
Experienced transition from car to active or public modes, t0 to t1		−0·31 (−0·48 to −0·13)	0·0005	−0·32 (−0·49 to −0·15)	0·0002	−0·30 (−0·47 to −0·13)	0·0005
Baseline BMI				−0·07 (−0·09 to −0·06)	<0·0001	−0·08 (−0·09 to −0·07)	<0·0001
Age (years)				−0·01 (−0·02 to −0·00)	0·013	−0·01 (−0·02 to −0·00)	0·0093
Sex							
	Male			0		0	
	Female			−0·03 (−0·14 to 0·08)	0·60	−0·03 (−0·14 to 0·08)	0·59
Ethnicity							
	White British			0		0	
	Other white background			0·06 (−0·16 to 0·28)	0·58	0·03 (−0·19 to 0·25)	0·79
	South Asian			−0·12 (−0·70 to 0·47)	0·70	−0·19 (−0·76 to 0·39)	0·53
	Black Caribbean			0·60 (−0·46 to 1·66)	0·27	0·50 (−0·54 to 1·54)	0·35
	Black African			−0·46 (−1·47 to 0·55)	0·37	−0·26 (−1·26 to 0·73)	0·61
	Chinese			−0·32 (−1·26 to 0·62)	0·51	−0·15 (−1·07 to 0·77)	0·75
	Mixed background			−0·81 (−1·66 to 0·04)	0·060	−0·78 (−1·62 to 0·06)	0·070
	Other ethnic background			−0·34 (−0·98 to 0·29)	0·29	−0·37 (−1·00 to 0·25)	0·24
Gross annual household income							
	<£18 000			0		0	
	£18 000–£30 999			−0·16 (−0·42 to 0·10)	0·24	−0·20 (−0·46 to 0·06)	0·13
	£31 000–£51 999			−0·21 (−0·46 to 0·04)	0·11	−0·23 (−0·49 to 0·02)	0·070
	£52 000–£100 000			−0·18 (−0·44 to 0·08)	0·18	−0·22 (−0·48 to 0·05)	0·11
	>£100 000			−0·11 (−0·42 to 0·21)	0·51	−0·15 (−0·47 to 0·16)	0·34
Gross annual household income category change, t0 to t1							
	Stable			0		0	
	Decrease			−0·05 (−0·20 to 0·10)	0·53	−0·06 (−0·21 to 0·09)	0·44
	Increase			−0·10 (−0·25 to 0·05)	0·18	−0·11 (−0·26 to 0·03)	0·13
Highest educational qualification at baseline							
	College or university degree			0		0	
	A levels or equivalent			0·17 (0·00 to 0·34)	0·051	0·19 (0·02 to 0·36)	0·025
	O levels/GCSEs or equivalent			0·05 (−0·10 to 0·20)	0·51	0·07 (−0·08 to 0·22)	0·35
	CSEs or equivalent			0·20 (−0·06 to 0·45)	0·13	0·19 (−0·06 to 0·44)	0·15
	NVQ, HND, HNC, or equivalent			0·06 (−0·18 to 0·29)	0·65	0·11 (−0·12 to 0·35)	0·36
	Other professional qualifications			0·34 (0·07 to 0·62)	0·014	0·35 (0·08 to 0·62)	0·012
	None of the above qualifications			0·04 (−0·26 to 0·35)	0·79	0·05 (−0·25 to 0·35)	0·74
Self-rated health transition between t0 and t1							
	Stable good health					0	
	Good to poor health					0·73 (0·53 to 0·92)	<0·0001
	Poor to good health					−0·66 (−0·90 to −0·42)	<0·0001
	Stable poor health					0·46 (0·28 to 0·63)	<0·0001
Manual work status at baseline							
	Non-manual work					0	
	Manual work					−0·07 (−0·25 to 0·12)	0·47
Change in manual work status between t0 and t1							
	Stable					0	
	Transition to non-manual work					0·19 (−0·06 to 0·44)	0·14
	Transition to manual work					0·08 (−0·17 to 0·33)	0·52
Days per week of ≥10 min moderate physical activity						0·00 (−0·03 to 0·03)	0·98
Change in days per week of ≥10 min moderate activity, t0 to t1							
	Stable					0	
	Decrease					0·16 (0·01 to 0·30)	0·031
	Increase					−0·18 (−0·32 to −0·04)	0·011
Job involves standing or walking at baseline							
	Never/rarely					0	
	Sometimes					0·02 (−0·13 to 0·16)	0·84
	Usually/always					−0·05 (−0·23 to 0·14)	0·61
Change in occupational standing or walking levels, t0 to t1							
	Stable					0	
	Decrease					0·19 (0·02 to 0·36)	0·032
	Increase					−0·18 (−0·35 to −0·01)	0·036

**Table 4:** Nested multivariate linear regression models testing whether experiencing a transition from active or public mode commuting to car commuting between baseline and follow independently predicted body-mass index change (n=1735)

		**Model 1**		**Model 2**		**Model 3**	
		Coefficient (95% CI)	p value	Coefficient (95% CI)	p value	Coefficient (95% CI)	p value
Stable active or public modes user		0		0		0	
Experienced transition from active or public modes to car, t0 to t1		0·31 (0·13 to 0·49)	0·0009	0·36 (0·17 to 0·54)	0·0001	0·32 (0·13 to 0·50)	0·0008
Baseline BMI				−0·06 (−0·08 to −0·04)	<0·0001	−0·07 (−0·08 to −0·05)	<0·0001
Age (years)				0·01 (0·00 to 0·02)	0·168	0·01 (0·00 to 0·02)	0·206
Sex							
	Male			0		0	
	Female			−0·03 (−0·19 to 0·13)	0·735	−0·01 (−0·16 to 0·15)	0·936
Ethnicity							
	White British			0		0	
	Other white background			−0·07 (−0·38 to 0·23)	0·636	−0·10 (−0·40 to 0·20)	0·520
	South Asian			0·30 (−0·67 to 1·28)	0·542	0·26 (−0·72 to 1·23)	0·606
	Black Caribbean			0·69 (−0·54 to 1·91)	0·270	0·64 (−0·58 to 1·86)	0·303
	Black African			−1·02 (−2·04 to 0·01)	0·052	−1·09 (−2·11 to −0·070)	0·036
	Chinese			0·44 (−0·78 to 1·66)	0·482	0·48 (−0·74 to 1·69)	0·443
	Mixed background			−0·71 (−1·68 to 0·27)	0·156	−0·66 (−1·64 to 0·31)	0·181
	Other ethnic background			0·18 (−0·72 to 1·08)	0·696	0·07 (−0·83 to 0·97)	0·876
Gross annual household income							
	<£18 000			0		0	
	£18 000–£30 999			−0·12 (−0·42 to 0·18)	0·426	−0·07 (−0·37 to 0·23)	0·651
	£31 000–£51 999			−0·10 (−0·40 to 0·20)	0·520	−0·06 (−0·37 to 0·24)	0·673
	£52 000–£100 000			−0·09 (−0·40 to 0·22)	0·581	−0·03 (−0·35 to 0·28)	0·849
	>£100 000			0·12 (−0·29 to 0·53)	0·574	0·19 (−0·22 to 0·60)	0·367
Gross annual household income category change, t0 to t1							
	Stable			0		0	
	Decrease			−0·30 (−0·52 to −0·08)	0·0071	−0·30 (−0·52 to −0·08)	0·0082
	Increase			−0·05 (−0·26 to 0·17)	0·661	−0·07 (−0·28 to 0·15)	0·540
Highest educational qualification at baseline							
	College or university degree			0		0	
	A levels or equivalent			0·15 (−0·09 to 0·38)	0·221	0·12 (−0·11 to 0·36)	0·310
	O levels/GCSEs or equivalent			0·20 (−0·03 to 0·43)	0·084	0·18 (−0·05 to 0·41)	0·125
	CSEs or equivalent			0·64 (0·21 to 1·07)	0·0033	0·57 (0·14 to 1·00)	0·0094
	NVQ, HND, HNC, or equivalent			0·14 (−0·24 to 0·53)	0·458	0·03 (−0·36 to 0·42)	0·881
	Other professional qualifications			0·43 (−0·05 to 0·91)	0·080	0·44 (−0·04 to 0·92)	0·072
	None of the above qualifications			0·17 (−0·33 to 0·67)	0·505	0·07 (−0·44 to 0·57)	0·801
Self-rated health transition between t0 and t1							
	Stable good health					0	
	Good to poor health					0·55 (0·27 to 0·83)	0·0001
	Poor to good health					−0·46 (−0·79 to −0·14)	0·0055
	Stable poor health					0·17 (−0·12 to 0·46)	0·244
Manual work status at baseline							
	Non-manual work					0	
	Manual work					0·19 (−0·08 to 0·47)	0·171
Change in manual work status between t0 and t1							
	Stable					0	
	Transition to non-manual work					−0·10 (−0·49 to 0·29)	0·623
	Transition to manual work					−0·39 (−0·79 to 0·00)	0·049
Days per week of ≥10 min moderate physical activity						−0·01 (−0·05 to 0·03)	0·596
Change in days per week of ≥10 min moderate activity, t0 to t1							
	Stable					0	
	Decrease					0·10 (−0·10 to 0·29)	0·333
	Increase					−0·05 (−0·25 to 0·16)	0·660
Job involves standing or walking at baseline							
	Never/rarely					0	
	Sometimes					0·06 (−0·15 to 0·27)	0·573
	Usually/always					0·03 (−0·26 to 0·31)	0·849
Change in occupational standing or walking levels, t0 to t1							
	Stable					0	
	Decrease					0·11 (−0·15 to 0·36)	0·398
	Increase					0·00 (−0·24 to 0·23)	0·982
