# Correction to *Lancet Public Health* 2016; 1: e46–55

**DOI:** 10.1016/S2468-2667(16)30042-1

**Published:** 2016-12-15

*Flint E, Webb E, Cummins S. Change in commute mode and body-mass index: prospective, longitudinal evidence from UK Biobank. Lancet Public Health 2016;* 1: *e46–55*—In the Research in Context panel of this Article, switching to active commuting should have been associated with a decrease in body mass index and switching to passive commuting associated with an increase. These changes have been made to the online version as of Dec 14, 2016.

